# Noble Metal Nanoparticles Applications: Recent Trends in Food Control

**DOI:** 10.3390/bioengineering6010010

**Published:** 2019-01-21

**Authors:** Giuliana Vinci, Mattia Rapa

**Affiliations:** Laboratory of Commodity Sciences, Department of Management, Sapienza University of Rome, via del Castro Laurenziano 9, 00161 Rome, Italy

**Keywords:** noble metal nanoparticles, food control, AuNPs, AgNPs, PtNPs, contaminants, nutrients, bioactive compounds

## Abstract

Scientific research in the nanomaterials field is constantly evolving, making it possible to develop new materials and above all to find new applications. Therefore, nanoparticles (NPs) are suitable for different applications: nanomedicine, drug delivery, sensors, optoelectronics and food control. This review explores the recent trend in food control of using noble metallic nanoparticles as determination tools. Two major uses of NPs in food control have been found: the determination of contaminants and bioactive compounds. Applications were found for the determination of mycotoxins, pesticides, drug residues, allergens, probable carcinogenic compounds, bacteria, amino acids, gluten and antioxidants. The new developed methods are competitive for their use in food control, demonstrated by their validation and application to real samples.

## 1. Introduction 

Nanoparticles (NPs) are characterized by different properties, depending on their size and typical structural form. A high surface/volume ratio gives NPs different properties compared to the same materials on a macroscopic scale, making unique applications possible [1]. The changes are due to quantum effects: variation in the electronic structure, a high number of superficial atoms, an increase in unsaturated bonds (dangling bonds), and variations in the band gap [2]. The nano size of NPs is gained by controlled synthesis in order to obtain nanomaterials for specific applications [3]. This allows one to obtain nanostructures with specific morphologies, controlled structures and functional properties. A large quantity of nanoparticles exist: metallic NPs [4], polymeric NPs [5], magnetic NPs [6], etc. NPs can also have different functionalization, such as hydrophilic or hydrophobic ones [7], which strongly determines their applications. Therefore, NPs can be suitable for different applications: nanomedicine [8], drug delivery [9,10], sensors [11,12], optoelectronics [13,14] and food control [15]. 

In this framework, scientific research is constantly evolving, making it possible to synthetize new materials and find new applications [16].

The exclusive physical–chemical properties of noble metal NPs (NMNPS) gives them high multifunctionality [17]. Noble metallic nanoparticles, such as AuNPs, AgNPs, PtNPs, offer high stability, easy chemical synthesis and tuneable surface functionalization [18]. 

NMNPs are involved in bioactive compounds and contaminant sensing, based on different methods such as colorimetry, immunoassays, Raman spectroscopy and sensors. This review deals with the recent trend of the application of noble metallic nanoparticles in food control [19]. Contaminants, nutrients and bioactive molecule detection in food are highlighted. In this framework, many scientific papers were found and in this systematic review the use of noble metal nanoparticles, such as gold nanoparticles (AuNPs), silver nanoparticles (AgNPs) and platinum trattare nanoparticles (PtNPs), were analysed as determination tools in food. Two major uses of NPs in food control have been found: the determination of contaminants (such as mycotoxins, pesticides, etc.) and bioactive compounds (nutrients, antioxidant compounds, proteins, etc.).

## 2. Contaminants Determination

Contaminants are chemical or biological substances, not intentionally added in food, that may be present as result of the various stages of their production, processing or transport. They can also occur as a result of environmental contamination. Contaminants can represent a risk to human and animal health. Therefore, to ensure food safety, contaminants determinations is a necessary step [20]. The determination of these species in food is usually conducted with traditional methods, such as spectrophotometric or chromatographic ones. In this framework, noble metal NPs are widely used as an alternative method. The main applications, reported in literature, are for determining contamination from mycotoxins (Table 1), drug residues and allergens (Table 2), probable carcinogenic compounds (Table 3), pesticides (Table 4) and bacteria (Table 5).

### 2.1. Mycotoxins

The main application of noble metal nanoparticles is mycotoxins determination. Mycotoxins (aflatoxins, trichothecenes, fumonisins, etc.), produced by mould and other microscopic species, are responsible for acute and chronic toxicity. Mycotoxins can be grouped into four categories, based on their mechanism of action: cytotoxic poisons; neurotoxins; gastrointestinal irritants and toxins that cause symptoms only in association with the ethyl alcohol consumption [21]. Mycotoxins usually found in food are accumulated as secondary mould products of the genera *Aspergillus*, *Penicillium* and *Fusarium* [22].

AuNPs in this application are used by electrochemical determination and the colorimetric method, based on the surface plasmon resonance (SPR) changes, by alteration in NPs size or aggregation [23].

A recent publication deals with the detection of aflatoxin B1 by using an immunosensor [24]. AuNPs of different size and origin were immobilized on the sensor surface, in order to increase the work surface and then enhance the signal. The developed method was compared with HPLC reference measurements with good results and then used in the detection of aflatoxin in paprika matrix.

Alternariol monomethyl ether (AME), a compound that possesses mutagenicity and carcinogenicity properties, is usually found in a wide range of vegetables, fruit and grains. An AME determination method was developed in a recent paper [25]. The colorimetric immunosensor method is based on the aggregation of AuNPs, and functionalized with a monoclonal antibody that competitively binds AME molecules in samples. This method is simple, rapid, and highly sensitive, with a competitive limit of detection (0.16 ng/mL) and recoveries (from 80.6% to 90.7%). Apart from this work, many papers were found on the use of AuNPs for colorimetric assays, such as the simultaneous detection of aflatoxin B1 and type-B fumonisins in wheat and wheat products [26], the determination of strychnine in nux-vomica seeds [27], and the detection of cyanide and linamarin in food [28,29,30,31]. Furthermore, binary systems of AuNPs and other noble metals are used to detect some toxins. Platinum-coated gold nanorods (AuNR@Pt) were used for fast and accurate detection of staphylococcal enterotoxin B through the complementary DNA fragment of toxin aptamer immobilization [32] and Au(core)@Au-Ag(shell) nanogapped nanostructures, for the Ochratoxin A detection, through an ultrasensitive surface-enhanced Raman scattering (SERS) aptasensor [33].

### 2.2. Drug Residues and Allergens

The consumer’s exposure to veterinary drugs represents a health hazard. Veterinary drugs used in food-producing animals can generate residues in animal derived products, widely consumed, such as meat, milk, eggs and honey. In this way a lot of AuNPs application were found for their determination in food matrices. A method was found for the on-site visual detection of amantadine residues in poultry [34]. This interesting paper deals with the possibility of amplifying the signal of the designed immunoassay by combining it with the conventional indirect competitive enzyme-linked immunosorbent assay, Fenton reaction-regulated oxidation of cysteine, and gold nanoparticle aggregation. The cascade reaction enhanced the assay sensitivity and led to a pronounced colour change from red to dark purple, which could be easily distinguished with the naked eye even at approximately 1 μg/Kg of poultry muscle. This immunoassay could be simply applicable for on-site detection in food control. Some methods have also been developed to detect antibiotics abused in animal husbandry, that could be found as residues in animal-derived food. These methods, both based on AuNP aggregation, have been developed to determinate aminoglycoside antibiotics [35] and ceftriaxone [36] in animal-origin foods, such as milk, eggs and meat. The spectral changes induced by AuNP aggregation were analysed with a pattern recognition technique (i.e., Cluster Analysis) for aminoglycoside antibiotics and with spectrophotometric surface plasmon resonance (SPR) band shift for ceftriaxone.

For allergens, a voltammetry biosensor consisting of an AuNP-coated screen-printed carbon electrode combined with sandwich immunoassay to detect peanut allergens *Ara h 1* and *Ara h 6* in food samples was developed [37,38]. The conjugation of AuNPs with monoclonal antibodies captures the analyte proteins, then, an enzymatic reaction is carried out and the electrochemical stripping current of this reaction corresponds to the peanut allergen amount in the food samples.

### 2.3. Probable Carcinogenic Compounds 

Probable carcinogenic compounds are substances that should be considered carcinogenic to humans. They have enough elements to believe that human exposure to the substance causes the development of tumours, in general, based on adequate long-term studies carried out on animals or other specific information [39]. Unfortunately, there are many compounds that derive from reactions that occur in food production (such as acrylamide or azodicarbonamide) or from preservation techniques (nitrites and melamine) [40]. A colorimetric assay for acrylamide in food was developed by Shi et al., based on acrylamide copolymerization. AuNPs were modified with a thiolated propylene amide poly (ethylene glycol) and the method is based on colour changes induced by an increase in the distance between gold nanoparticles (AuNPs). This method can be used for a rapid sensing of acrylamide traces in food, with a 0.2 nM limit of detection and a lower relative error (RSD%) compared to the accepted HPLC method [41]. Also, for azodicarbonamide (ADA) in flour products, a colorimetric method has been developed [42], based on glutathione (GSH)-induced gold nanoparticle (AuNPs) aggregation. This method, with high recoveries (91–104%) and low RSD% (<6%), can be used to detect 38.3 ppb of ADA by naked eye observation and 26.7 ppb of ADA by spectrophotometry, both lower than the ADA limitation in flour (45 mg/kg).

Due to preservation techniques, melamine and nitrites are widely present in food. An assay for melamine detection with AgNPs was recently developed by Jigyasa et al., by the interaction of melamine with Ag^+^ ions. At low concentrations of melamine, pale red coloured solution was obtained due to the formation of an aggregated mass of AgNPs, whereas, at high concentrations of melamine, colourless solution was obtained, indicating disruption in the synthesis of AgNPs [43]. For nitrites a gold nanoparticle/poly(methylene blue) (GNP/PMB)-modified pencil graphite electrode (PGE) was used [44]. This method was applied to commercial sausage and mineral water samples, where a linear relationship was observed in the concentration range of 5–5000 μM. Indeed, the limit of detection (0.314 μM, S/N = 3) and reproducibility (RSD = 2.38% for N = 10) also gave good results.

### 2.4. Pesticides

The residual pesticides in fruit and vegetables are one of the major food safety concerns for consumers. In this context, many NP-based analytical methods to sense pesticide residues in foods have recently been developed. Two pesticides have been detected with surface enhanced Raman spectroscopy (SERS) coupled with AuNPs for atrazine in apple juice [45] and with core-shell Au@Ag nanoparticle aggregates for difenoconazole in grapes [46]. Another potential application in food safety with SERS coupled methods was developed by Ma et al. In this work, a possible use of AgNPs/GO (Graphene Oxide) paper for pesticides determination in food was investigated with good results [47].

### 2.5. Bacteria

Bacteria determination is an essential step in food safety assessment. The absence of certain bacteria in food is necessary to eat food safely, because some bacterial species have negative effects on human health. They can cause diarrhoea, typhoid fever, haemolytic uraemic syndrome and haemorrhagic colitis. All the papers found on literature review deal with an AuNP-based method. One paper detected some bacteria, such as *Escherichia coli* and *Salmonella*, in water and milk, by lateral flow immunoassay [48]. The antibodies were immobilized on AuNPs and lateral flow immunoassays were carried out with 100% specificity. This method was able to detect *Escherichia coli* in water and milk samples as low as 7.8 × 10^5^ and 3 × 10^6^ CFU/mL (colony-forming unit/volume), respectively, and as low as 3 × 10^8^ and 3 × 10^7^ CFU/mL in water and milk samples, respectively, for *Salmonella typhi*. Other papers were found based on a colorimetric assay for sensing *Salmonella* in milk and shrimp [49], *Listeria* in lettuce [50] and *Escherichia coli* and *Salmonella* in water and milk [51]. All these sensing methods gave good results and their application in food control is easy, due to the simple colour change of the solution after the AuNP interaction. A noteworthy work was found on *Salmonella* detection in spiked food [52] by applying a microfluidic system. This tool was noticeable for having a low-cost and in situ detection, with a very low LOD (limit of detection) of 10 CFU/25 g. This methodology has represented a starting point for future works on detection of different pathogens.

## 3. Nutrients and Bioactive Compounds

Analysis of food continuously requires development of more robust, efficient, sensitive, and cost-effective analytical methodologies to determinate nutrients and bioactive compounds of food [53]. These determinations guarantee the safety, quality and traceability of food, in compliance with legislation and consumers’ demands. In this framework, the use of noble metal nanoparticles was recently suggested as an alternative to classical methods of determination [54]. NPs could improve the analytical accuracy, precision, detection limits, and sample quantity, thereby expanding the practical range of food applications [2]. As found in the literature, NP applications in this context were amino acid, gluten (gliadin) (Table 6) and antioxidant compound (Table 7) determination.

### 3.1. Amino Acids

Amino acids (AAs), the fundamental units of proteins, are usually classified as essential AAs, semi-essential AAs and non-essential AAs. The essential ones must be assumed by diet, as the human body is not able to synthetize them. An amperometric immunosensor for the detection of monosodium glutamate (MSG) was recently reported by Devi et al. In this sensor, the anti-glutamate antibody was immobilized on to the sensor surface, composed of a carbon electrode modified with gold nanoparticles decorated on a molybdenum disulfide/chitosan (Au@MoS2/Ch) nanocomposite. This method was validated and showed limit of detection and limit of quantification values of 0.03 and 0.1 µM, respectively, with a detection range of 0.05–200 µM [54]. A method based on optical absorption was developed by Li et al. for the rapid detection of L-cysteine with gold nanoparticles on a graphene oxide substrate, as sensitive nanoprobes. This method uses a smartphone-based system with multimode analysis of red-green-blue (RGB), hue-saturation-value (HSV), hue-saturation-lightness (HSL), and cyan-magenta-yellow-black (CMYK) values [55]. These features were proposed for the investigation of interactions between the nanoprobes and L-cysteine, with high applicability in food control.

### 3.2. Gluten

Gluten is a protein complex, typical of some cereals, characterized by two proteins: glutenin and gliadin [56]. The latter is the main protein responsible for the phenomena of allergic reactions [57], and the main NP applications are based on this. Many immunosensors based on modified AuNPs, to detect gliadin in food samples, have been recently developed [58,59], and are also based on the DNA recognition [60]. 

### 3.3. Antioxidants

Antioxidants, metabolites of many plants, are found in a ubiquitous way in food, especially in vegetables, and they are one of the most important groups of natural compounds [61,62]. They have anti-microbial and anticarcinogenic effects and above all a high antioxidant activity, demonstrated in vivo and in vitro experiments [63]. In addition, their possible effects against cardiovascular diseases and neurodegenerative disorders have recently been demonstrated [64,65]. Considered as “molecular markers” of food quality, many articles are published every year about their determination. Some new antioxidant determination methods involve the use of NMNPs in two areas: the phenolic compound (principal class of antioxidant) determination and the antioxidant activity assays.

For the phenolic compound determination, a colorimetric assay was presented by Della Pelle et al. This method was based on the synthesis of AuNPs by the endogenous phenolic compound present in fat matrix. The phenolic compound concentration was related to the AuNP synthesis, controlled by the surface plasmon resonance [66]. The method was validated in comparison to the total phenolic compound determination, operated by Folin–Ciocalteu (FC) reagent. The same reference method was also used in another paper, which deals with the use of functionalized AuNPs for the phenolic compound extraction from olive oil. This rapid and sustainable method was optimized by applying a response surface methodology, with the construction of a central composite design (CCD) of some variables such as the AuNPs amount or the stirring contact time between oil and NPs [67].

Another colorimetric assay for analysis of 20 antioxidants in beverages, such as tea and lemon juice, was also developed by the aggregation or morphological changes of AuNPs and AgNPs [68]. Many applications of NPs were found for the antioxidant capacity (AOC) assay, in differences matrices. A sensitive AgNPs spectrophotometric method for antioxidant capacity has been developed, based on the ability of natural polyphenols to reduce Ag(I) and stabilize the produced Ag(0)NPs [69]. Other colorimetric methods have been developed for the AOC assays: AuNPs based in tea extract [70] and olive oil [71]; PtNPs [72] for the methanolic and aqueous extracts of teas and infusions.

## 4. Conclusions

This work has explored the recent trend in food control of using the noble metallic nanoparticles as determination tools. NPs are suitable for different applications and in food control are commonly used to detect specific compounds. In this review, the possibility of NMNPs use for contaminants, nutrients and bioactive molecule detection are highlighted. Applications were found for the determination of mycotoxins, pesticides, drug residues, allergens, probable carcinogenic compounds, bacteria, amino acids, gluten and antioxidants. The new developed methods are competitive for their use in food control, demonstrated by their validation and application to real samples. 

## Figures and Tables

**Table 1:** Mycotoxin determination in food by Noble Metal NPs (* various).

Matrix	Mycotoxin	Molecular Weight	Chemical Structure	Nanoparticles	Method	References
Paprika	Aflatoxin B1	312.28 g/mol	C_17_H_12_O_6_	AuNPs	Immunosensor	[24]
Fruits, Vegetables, Grains	Alternariol Monomethyl Ether	272.256 g/mol	C_15_H_12_O_5_	AuNPs	Colorimetric Immunosensor	[25]
Wheat, Wheat Products	Type-B Fumonisins	721.84 g/mol	C_34_H_59_NO_15_	AuNPs	Colorimetric Assays	[26]
Nux-Vomica Seeds	Strychnine	334.42 g/mol	C_21_H_22_N_2_O_2_	AuNPs	Colorimetric Assays	[27]
Vegetables	Cyanide Linamarin	26.02 g/mol 247,248 g/mol	CN^−^ C_10_H_17_NO_6_	AuNPs	Colorimetric Assays	[28,29,30,31]
Milk	Enterotoxin B	30,000 ± 1000 g/mol	*	AuNR@Pt	DNA Fragment of Toxin Aptamer Immobilization	[32]
Wine	Ochratoxin A	403.813 g/mol	C_20_H_18_ClNO_6_	Au(core)@Au-Ag(shell)	Surface-Enhanced Raman Scattering	[33]

**Table 2:** Drug residues and allergens determination in food by noble metal NPs (* various).

Matrix	Drug residues and Allergens	Molecular Weight	Chemical Structure	Nanoparticles	Method	References
Poultry	Amantadine	151.253 g/mol	C_10_H_17_N	AuNPs	Immunoassay	[34]
Milk	Aminoglycoside antibiotics	*	*	AuNPs	Pattern Recognition	[35]
Milk, Meat	Ceftriaxone	554.58 g/mol	C_18_H_18_N_8_O_7_S_3_	AuNPs	NPs Aggregation	[36]
Cookies, Chocolate	*Ara h 1, Ara h 6* (peanut allergens)	500–600 kDa	*	AuNPs	Immunoassay	[37,38]

**Table 3:** Probable carcinogenic compound determination in food by noble metal NPs.

Matrix	Probably Carcinogenic Compounds	Molecular Weight	Chemical Structure	Nanoparticles	Method	References
Potato Chips, Cookies	Acrylamide	71.08 g/mol	C_3_H_5_NO	AuNPs	NPs Aggregation	[41]
Flour Products	Azodicarbonamide	116.08 g/mol	C_2_H_4_N_4_O_2_	AuNPs	NPs Aggregation	[42]
Milk	Melamine	126.12 g/mol	C_3_H_6_N_6_	AgNPs	Colorimetric Assays	[43]
Sausage, Water	Nitrites	46.01 g/mol	NO_2_^−^	AgNPs	Sensoristic	[44]

**Table 4:** Pesticides determination in food by Noble Metal NPs (* various).

Matrix	Pesticides	Molecular Weight	Chemical Structure	Nanoparticles	Method	References
Apple Juice	Atrazine	215.68 g/mol	C_8_H_14_ClN_5_	AuNPs	NPs Aggregation	[45]
Grapes	Difenoconazole	406.263 g/mol	C_19_H_17_Cl_2_N_3_O_3_	Au@AgNPs	NPs Aggregation	[46]
Fruit, Vegetables	Various Pesticides	*	*	AgNPs	Colorimetric Assays	[47]

**Table 5:** Bacteria determination in food by noble metal NPs.

Matrix	Bacteria	Nanoparticles	Method	References
Water, Milk	*Escherichia coli, Salmonella*	AuNPs	Immunoassays	[48]
Milk, Shrimp	*Salmonella*	AuNPs	Colorimetric Assays	[49]
Lettuce	*Listeria*	AuNPs	Colorimetric Assays	[50]
Water, Milk	*Escherichia coli, Salmonella*	AuNPs	Colorimetric Assays	[51]
Chicken, Turkey, Egg Products	*Salmonella*	AuNPs	Microfluidic	[52]

**Table 6:** Gluten determination in food by noble metal NPs.

Matrix	Compounds	Molecular Weight	Chemical Structure	Nanoparticles	Method	References
Flours (Mile, Chestnut, Chickpeas, Quinoa, Potato)	Gliadin	631.687 g/mol	C_29_H_41_N_7_O_9_	AuNPs	Immunoassay	[58]
Cereals	Gliadin	631.687 g/mol	C_29_H_41_N_7_O_9_	AuNPs	Immunoassay	[59]
Wheat, Barley, Oat, Rice, Foxtail Millet, Corn, Buckwheat, Soybean, Rye	Gliadin	631.687 g/mol	C_29_H_41_N_7_O_9_	AuNPs	DNA recognition	[60]

**Table 7:** Antioxidant determination in food by noble metal NPs.

Matrix	Determination	Nanoparticles	Method	References
Fat matrix	Phenolic Compounds	AuNPs	SPR	[66]
Olive oil	Phenolic Compounds	AuNPs	Extraction	[67]
Tea, Lemon Juice	Phenolic Compounds	AuNPs, AgNPS	Colorimetric Assays	[68]
Tea	Antioxidant Capacity	AgNPs	NPs synthesis - SPR	[69]
Tea	Antioxidant Capacity	AuNPs	Colorimetric Assays	[70]
Olive Oil	Antioxidant Capacity	AuNPs	Colorimetric Assays	[71]
Tea, Infusions	Antioxidant Capacity	PtNPs	Colorimetric Assays	[72]
